# Objectives and Methods of Iron Chelation Therapy

**DOI:** 10.1155/S1565363303000128

**Published:** 2003

**Authors:** C. Hershko, A. Abrahamov, A. M. Konijn, W. Breuer, I. Z. Cabantchik, P. Pootrakul, G. Link

**Affiliations:** 1 Department of Medicine Shaare Zedek Med Ctr. Hebrew University of Jerusalem Israel; 2 Department of Human Nutrition and Metabolism Hebrew University of Jerusalem Israel; 3 DeparOnem of Biological Chemistry Hebrew University of Jerusalem Israel; 4 Mahidol University Bangkok Thailand

## Abstract

Recent developments in the understanding of the molecular control of iron homeostasis provided novel insights into the mechanisms responsible for normal iron balance. However in chronic anemias associated with iron overload, such mechanisms are no longer sufficient to offer protection from iron toxicity, and iron chelating therapy is the only method available for preventing early death caused mainly by myocardial and hepatic damage. Today, long-term deferoxamine (DFO) therapy is an integral part of the management of thalassemia and other transfusion-dependent anemias, with a major impact on well-being and survival. However, the high cost and rigorous requirements of DFO therapy, and the significant toxicity of deferiprone underline the need for the continued development of new and improved orally effective iron chelators. Within recent years more than one thousand candidate compounds have been screened in animal models. The most outstanding of these compounds include deferiprone (L1); pyridoxal isonicotinoyl hydrazone (PIH) and; bishydroxy- phenyl thiazole. Deferiprone has been used extensively as a substitute for DFO in clinical trials involving hundreds of patients. However, L1 treatment alone fails to achieve a negative iron balance in a substantial proportion of subjects. Deferiprone is less effective than DFO and its potential hepatotoxicity is an issue of current controversy. A new orally effective iron chelator should not necessarily be regarded as one displacing the presently accepted and highly effective parenteral drug DFO. Rather, it could be employed to extend the scope of iron chelating strategies in a manner analogous with the combined use of medications in the management of other conditions such as hypertension or diabetes. Coadministration or alternating use of DFO and a suitable oral chelator may allow a decrease in dosage of both drugs and improve compliance by decreasing the demand on tedious parenteral drug administration. Combined use of DFO and L1 has already been shown to result in successful depletion of iron stores in patients previously failing to respond to single drug therapy, and to lead to improved compliance with treatment. It may also result in a “shuttle effect” between weak intracellular chelators and powerful extracellular chelators or exploit the entero-hepatic cycle to promote fecal iron excretion. All of these innovative ways of chelator usage are now awaiting evaluation in experimental models and in the clinical setting.


### Objectives and Methods of Iron Chelation Therapy

C Hershko[1], A Abrahamov[1], AM Konijn[2], W Breuer[3] IZ Cabantchik[3], P Pootrakul[4] and G Link[2]

[1]*Department of Medicine, Shaare Zedek Med Ctr.*

[2]*Department of Human Nutrition and Metabolism and*

[3]*Department of Biological Chemistry, Hebrew University of Jerusalem, Israel and*

[4]*Mahidol University, Bangkok, Thailand*

(Received November 12, 2002; Revised January 22, 2003; Accepted January 30, 2003)

**ABSTRACT**

Recent developments in the understanding of the molecular control of iron homeostasis provided novel insights into the mechanisms responsible for normal iron balance. However in chronic anemias associated with iron overload, such mechanisms are no longer sufficient to offer protection from iron toxicity, and iron chelating therapy is the only method available for preventing early death caused mainly by myocardial and hepatic damage. Today, long-term deferoxamine (DFO) therapy is an integral part of the management of thalassemia and other transfusion-dependent anemias, with a major impact on well-being and survival. However, the high cost and rigorous requirements of DFO therapy, and the significant toxicity of deferiprone underline the need for the continued development of new and improved orally effective iron chelators. Within recent years more than one thousand candidate compounds have been screened in animal models. The most outstanding of these compounds include deferiprone (L1); pyridoxal isonicotinoyl hydrazone (PIH) and; bishydroxy- phenyl thiazole. Deferiprone has been used extensively as a substitute for DFO in clinical trials involving hundreds of patients. However, L1 treatment alone fails to achieve a negative iron balance in a substantial proportion of subjects. Deferiprone is less effective than DFO and its potential hepatotoxicity is an issue of current controversy. A new orally effective iron chelator should not necessarily be regarded as one displacing the presently accepted and highly effective parenteral drug DFO. Rather, it could be employed to extend the scope of iron chelating strategies in a manner analogous with the combined use of medications in the management of other conditions such as hypertension or diabetes. Coadministration or alternating use of DFO and a suitable oral chelator may allow a decrease in dosage of both drugs and improve compliance by decreasing the demand on tedious parenteral drug administration. Combined use of DFO and L1 has already been shown to result in successful depletion of iron stores in patients previously failing to respond to

*Correspondence: C Hershko, Dept Medicine, Shaare Zedek Med Center, Jerusalem, Israel, P O Box 3235 , *phone* 972-2-6555567, *fax* 972-2-6555231; e-mail: hershko@sznc.org.il single drug therapy, and to lead to improved compliance with treatment. It may also result in a "shuttle effect" between weak intracellular chelators and powerful extracellular chelators or exploit the entero-hepatic cycle to promote fecal iron excretion. All of these innovative ways of chelator usage are now awaiting evaluation in experimental models and in the clinical setting.

### INTRODUCTION

Recent developments in the understanding of the molecular control of iron homeostasis provided novel insights into the mechanisms responsible for normal iron balance. However in chronic anemias associated with iron overload, such mechanisms are no longer sufficient to offer protection from iron toxicity, and iron chelating therapy is the only method available for preventing early death caused mainly by myocardial and hepatic damage. Although deferoxamine (DFO) has been available for treating transfusional iron overload from the early 1960s, the era of modern and effective iron chelating therapy started only 20 years ago with the introduction of subcutaneous DFO infusions by portable pumps. Today, long-term DFO therapy is an integral part of the management of thalassemia and other transfusion-dependent anemias, with a major impact on well-being and survival.

### PATHOPHYSIOLOGY OF IRON OVERLOAD IN THALASSEMIA:

The primary abnormality in thalassemia major is a wasteful, ineffective erythropoiesis resulting in a 10 to 15-fold expansion of the erythroid bone marrow /1/ and a drastic increase in hemoglobin catabolism. Iron accumulation is the consequence of blood transfusions as well as of increased iron absorption caused by erythropoietic activity. The role of increased iron absorption is illustrated by the severe iron overload encountered in patients with thalassemia intermedia who have never received blood transfusions. The combination of iron overload and increased outpouring of catabolic iron from the reticuloendothelial system overwhelms the iron carrying capacity of transferrin, resulting in the emergence of toxic non-transferrin bound plasma iron (NTBI).

Although our original description of a chelatable, low molecular weight plasma iron fraction in thalassemic patients with severe iron overload /2/ has been greeted with initial scepticism, its existence has been confirmed by many subsequent studies using a variety of methods. NTBI promotes the formation of free hydroxyl radicals and accelerates the peroxidation of membrane lipids in vitro /3/. Long-term treatment with DFO or deferiprone (L1) in thalassemic patients results in a marked decrease in their NTBI measurements /4/. More recently, Porter *et al.* /5/ have shown that plasma NTBI is removed by intravenous DFO therapy in a biphasic manner and that upon cessation of DFO infusion it reappears rapidly, lending support to the continuous, rather than intermittent, use of DFO in high risk patients. The rate of low molecular weight iron uptake by cultured rat heart cells is over 300-times that of transferrin iron /6/. Moreover, unlike transferrin-iron uptake which is inhibited at high tissue iron concentrations by down-regulation of transferrin receptor production, non-transferrin iron uptake is increased by high tissue iron /7/. Such uptake results in increased myocardial lipid peroxidation and abnormal contractility, and these effects are reversed by *in vitro* treatment with DFO /8/. Recognition of NTBI as a potentially toxic component of plasma iron in thalassemic siderosis has important practical implications for designing better strategies for the effective administration of DFO and other iron chelating drugs.

Without iron chelating therapy, the accumulation of iron will progress relentlessly and when about 20 grams of iron have been retained in the body, significant clinical manifestations of iron toxicity may be anticipated /9/. The most important complications of transfusional siderosis are cardiac, hepatic and endocrine disease. Pathologic findings in the heart include dilated, thickened ventricular walls with particularly heavy iron deposits in the ventricles, epicardium and papillary muscles. These cellular deposits induce increased membrane lipid peroxidation in the sarcolemma resulting in impaired Na,K,ATPase activity /10/, increased lysosomal fragility /11/ and, in particular, impaired mitochondrial inner-membrane respiratory chain activity /12/. It is possible to demonstrate early myocardial dysfunction in asymptomatic patients using MUGA scan /13/ or dobutamine stress echocardiography /14/. With progressive cardiac siderosis symptomatic heart failure and life-threatening arrhythmias will develop. Myocardial siderosis is the single most important cause of mortality in inadequately treated thalassemics.

### RESULTS OF LONG-TERM DFO TREATMENT

The introduction of deferoxamine (DFO, Figure 1) for the treatment of transfusional iron overload had a major impact on the survival and well-being of thalassemic patients. Although this has never been proven by prospective, randomized clinical studies, and such randomized studies are no longer justified ethically, the beneficial effects of long-term deferoxamine treatment are clearly demonstrated by comparison of treatment outcome with historical controls. Experience with long-term DFO therapy in thalassemic patients has been the subject of a number of excellent recent reviews /15,16/.

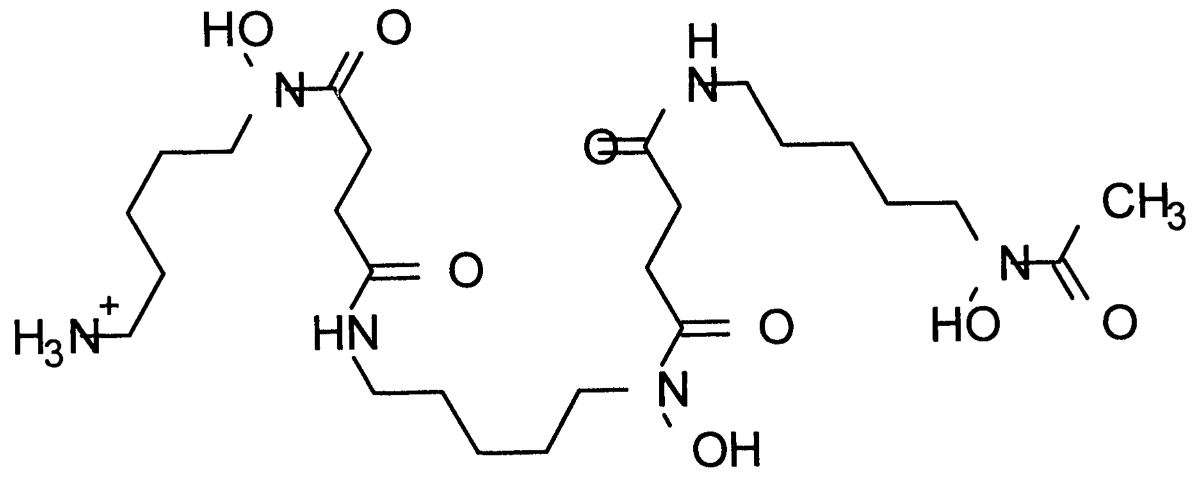

Fig. 1: Deferoxamine (DFO)

### Method of treatment:

The effect of treatment, measured by urinary iron excretion, is directly proportional to the severity of iron overload. Hence, treatment in subjects without iron overload will result in limited iron excretion. However, treatment should not be introduced too late if the objective is the prevention of iron toxicity. It should be started when serum ferritin levels reach about 1000 μg/l, which usually occurs after the first 10 or 20 transfusions /17/. DFO is infused via a thin s.c. needle inserted to the arm or abdomen nightly, connected to a portable pump over 8-12 h, 5 to 7 times per week at a daily dose of 20 to 60 mg/kg. A urinary iron excretion of 0.5 mg/kg/d is usually sufficient to ensure negative iron balance.

Although prolonged subcutaneous DFO infusion is universally recognized as the optimal method of treatment, twice daily *s.c.* injections may yield similar amounts of urinary iron /18/. A new delivery system for continuous DF infusion has been introduced by Baxter allowing continuous 48 h *s.c.* or continuous 24 h *i.v.* delivery for 7 days each week /19/. This technology allows effective removal of toxic free iron (NTBI) from the plasma, a significant decrease in serum ferritins within 4 weeks, and improves patient compliance compared to conventional *s.c.* DFO pumps. Compliance with the new disposable Baxter device allowing continuous DFO delivery for 48 or 120 hours has been quite satisfactory/(20/.

### Impact on survival:

The impact of desferrioxamine treatment on life expectancy is eloquently demonstrated by comparison of survival in well chelated versus poorly chelated patients. In a major study of 1127 thalassemic patients at 7 Italian teaching hospitals it was shown that 70% of patients born before 1970 and hence prior to the modern era of chelation survived to the age 20 y. compared to 88% in patients born after 1970 and therefore receiving effective chelation from an early age /9/. Most of the improvement in survival was attributed to decreased cardiac mortality. This cohort-of-birth related improvement in survival is reflected in a mirror-like inverse decrease in cardiac mortality, supporting the assumption that prevention of cardiac mortality is the most important beneficial effect of DFO therapy. A subsequent update on this group of patients /21/ has shown that the prevalence of heart failure and diabetes declines with subsequent birth cohorts. Conversely, hypothyroidism is becoming more frequent. Overall, diabetes was present in 5.4%, heart failure in 6.4%, thrombosis in 1.1%, HIV infection in 1.8%, and hypogonadism in 55% of those reaching pubertal age. This update on the cohort of patients described in earlier reports confirms the remarkable improvement in life expectancy comparing successive cohorts of thalassemic patients. This improvement is mainly due to decreased cardiac mortality. Conversely, mortality caused by other complications of thalassemia has not decreased. Improved survival in well chelated thalassemic patients has been reported in several other major studies from the U.K. and North America /22-25/. The increasing incidence with age of hypothyroidism, and the emergence of HIV as a significant cause of mortality has also been noticed in a group of thalassemic patients living in the New York area /26/.

The strongest direct evidence supporting the beneficial effect of DFO on hemosiderotic heart disease is the reversal of established myocardiopathy in some far-advanced cases. Earlier experience in hereditary hemochromatosis has shown that the myocardiopathy of iron overload is potentially curable by effective iron mobilization through phlebotomy. However, in transfusional hemosiderosis, the course of established myocardial disease was uniformly fatal and, until recently, believed to be non-responsive to iron chelating therapy. Several reports indicate that such patients may still be responsive to aggressive chelating treatment. Marcus *et al.* /27/ described first the reversal of established symptomatic myocardial disease in 3 of 5 patients by *continuous high-dose* (85-200 mg/kg/d) *i.v.* DFO therapy at the cost of severe reversible retinal toxicity. Reversal of symptomatic myocardiopathy has been reported by others, without significant drug toxicity /28,29/. Continuous 24-hour ambulatory intravenous infusion of DFO through central venous ports, using standard portable infusion pumps or the new Baxter delivery system /19,30/ is a very effective method for the rapid reversal of established hemosiderotic heart disease. In addition, it is an excellent tool for improving patient compliance allowing uninterrupted delivery of DFO and the effective depletion of very large iron stores.

### DEVELOPMENT OF ORALLY EFFECTIVE CHELATORS

In spite of the proven efficacy of desferrioxamine, not all patients are willing to cope with the rigorous requirements of the long-term use of portable pumps. In addition, the high cost of this treatment is a serious obstacle to its more widespread use. In view of these considerations, there is a great need for the development of alternative, orally effective iron chelating drugs. Within recent years more than one thousand candidate compounds have been screened in animal models. These efforts led to the identification of several interesting compounds, a few of which may be of possible clinical usefulness. The present discussion will be limited to the most outstanding of these compounds including deferiprone (L1); pyridoxal isonicotinoyl hydrazone (PIH) and bishydroxyphenyl thiazole.

### Deferiprone (L1).

Of all the new iron chelating drugs available today, only deferiprone has been used as a substitute for DFO in clinical trials involving many hundreds of patients. The family of 3-hydroxypyrid-4-one bidentate chelators, designed by Hider and Kontoghiorghes /31,32/ binds to iron in a 3:1 ratio. The most important compound of this family is 1,2-dimethyl-3-hydroxypyrid-4-one, (deferiprone or L1, Figure 2). Although clinical reports on the use of deferiprone in thalassemic and other patients have been published as early as 1987, detailed animal studies on toxicity and on pharmacokinetics have only become available subsequently. The pharmacology and clinical efficacy of deferiprone have been the subject of several reviews /33-36/.

*Animal toxicity*:

At a daily dose of 200 to 300 mg/kg, i.e. 3 to 4 times the dose recommended in man, given for several months, L1 caused bone marrow aplasia in mice, rats, dogs and monkeys, involution of lymphatic tissues and adrenal steatosis. These alterations were associated with high rates of mortality /37,38/. As shown below, clinical experience with L1 indicates that zinc deficiency is a common complication of L1 treatment /39/. Zinc is essential for the formation and function of the immune system. Zinc deprivation results in thymic atrophy, impaired function of macrophages and T cells, inability to respond to antigens and impaired

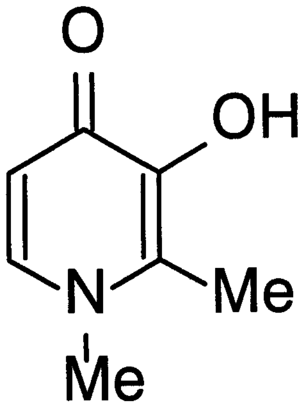

**Fig. 2:** 1,2-dimethyl-3-hydroxypyrid-4-one, (deferiprone or L1)

resistance to infection /40/. It also leads to gonadal atrophy and congenital malformations. Thus, it is quite likely that some of the most impressive toxic effects of deferiprone observed in animal studies of drug tolerability were caused by concurrent zinc deficiency and were not a direct consequence of deferiprone toxicity.

In view of animal toxicity data, in 1993 Ciba-Geigy announced the discontinuation of its efforts to develop deferiprone for clinical use /41/. This decision has been contested on the grounds that deferiprone should continue to be available for patients with severe transfusional iron overload who are unable or unwilling to use DFO /42/. It was also felt that toxicity of any chelator should be tested in iron-loaded and not in normal animals

*Clinical evaluation*:

The results of long-term iron chelating therapy with L1 in thalassemic patients /43-45/ have been summarized in several reviews /33-36/ and the combined experience of the 4 major European and Canadian groups pioneering the clinical use of L1 has been described in a report of the International Study Group for Oral Iron Chelators (ISGOIC) /39/. The study involved 84 patients, 74 with thalassemia major or intermedia, representing a total of 167 patient-years of L1 treatment. Compliance was rated as excellent in 48%, intermediate in 36% and poor in 16% of patients. On an L1 dose of 73 to 81 mg/kg/d, urinary iron excretion was stable, at around 0.5 mg/kg/d with no indication of a diminishing response with time. Serum ferritin showed a very steady decrease with time from an inital mean ±1SD of 4207 ±3118 to 1779 ±1154 after 48 months ($p < 0.001$). 17 patients abandoned L1 therapy. Major complications of L1 requiring permanent discontinuation of treatment included agranulocytosis /3/, severe nausea /4/, arthritis /2/ and persistent liver disfunction /1/. The remaining patients abandoned treatment because of low compliance /3/ and conditions unrelated to L1 toxicity. Lesser complications permitting continued L1 treatment included transient mild neutropenia /4/, zinc deficiency /12/ transient increase in liver enzymes /37/, moderate nausea /3/ and arthropathy /16/. There was no treatment-associated mortality. Two patients died, both while off treatment: one of hemosiderotic heart disease, and the other of pneumocystis carinii pneumonia with AIDS. This study demonstrates the efficacy of L1 in long-term use for the treatment of transfusional iron overload, but also underlines the complications associated with such treatment.

Subsequent experience in thalassemic patients on long-term L1 treatment has been reported by Olivieri *et al.* /46,47/, Hoffbrand *et al.* /48/ and Tondury *et al.* /49/ as well as in a report of a major multicenter study empoying the Apotex formulation of L1, involving 187 patients from Cagliari, Torino, Ferrara, Philadelphia and Toronto (the LA-02 study) /50/. All patients received a daily L1 dose of 75 mg/kg. The mean duration of treatment was 1.61 years for the study reported by Tricta, and 7.14 for that of Tondury, with the rest ranging from a mean of 3.28 to 4.60 years. Comparing these data with the ISGOIC study terminated in mid-1994 one can see that, with the exception of the Tondury study, there was little or no overlap with the patients participating in the earlier report, indicating a high turnover rate of the patient populations. Likewise, with the exception of the Olivieri and Tondury studies, there was no evidence of a consistent decrease in mean serum ferritins or liver iron concentrations comparing pre-treatment values with subsequent measurements. The proportion of patients in whom liver iron concentrations remained above 15 mg/g dry weight (identified in previous studies as the limit above which a significant risk of cardiac complications continues to exist) was 18 /49/, 37 /46/ and 59 /48/ percent. Agranulocytosis developed in 6 patients and transient neutropenia in 19. Although the mechanism of neutropenia in unknown, in some cases it is clearly an immune reaction to L1, as illustrated by the simultaneous development of agranulocytosis, systemic vasculitis, alterations in humoral and cell mediated immune function, and the presence of circulating immune complexes /51/.

In three of the above 4 reports the proportion of patients abandoning treatment has been specified . Of a total of 92 patients 36 (39%) discontinued L1 therapy. Six patients died. Of particular concern is the observation that four of the patients died with congestive heart failure due to iron overload, a complication which was shown previously to be prevented by effective deferoxamine therapy. Other important causes of L1 discontinuation were agranulocytosis or neutropenia (6 patients) arthropathy (5 patients) nausea (5 patients), or unsatisfactory response to L1 (8 patients) defined as low compliance (2), rising serum ferritins (4), request to resume DFO (1) and change of residence (1).

Thus, by comparison with the ISGOIC study summarizing L1 experience prior to June 1994, these recent reports indicate a higher rate of treatment discontinuation (39 *vs* 20 %), failure to decrease serum ferritin and liver iron concentrations to levels assuring significant cardioprotection in a substantial proportion of cases and, indeed the continued presence of cardiac mortality, a complication of transfusional iron overload which has already been largely eliminated by effective DFO treatment. A recent meta-analysis of nine clinical trials providing data on 129 iron overloaded patients /52/, has shown that after a mean of 16 months, 75% of patients with severe iron-overload had a decrease in serum ferritin as compared with baseline, and 51.8% achieved a negative iron balance. Other reports from Near-Eastern countries describe a high compliance rate in patients not previously compliant with DFO treatment, and a significant decrease in serum ferritin within the first year of deferiprone treatment /53/.

The failure to achieve a steady decrease in storage iron with L1 is explained by the difference in efficacy between the two drugs on a weight per weight basis. As shown by a metabolic balance study comparing combined urinary and fecal iron excretion in thalassemic patients receiving either 60-mg/kg DFO or 75 mg/kg *p.o.* L1, mean iron excretion on L1 was only 65% of that on DFO /54/. However, in some patients L1 was as effective, or better than L1.

Collectively, these data imply that oral L1 treatment alone will not ensure sufficient protection in all patients and, that close monitoring is required to identify patients in whom additional conventional chelating treatment with DFO is indicated. In patients with unsatisfactory response to deferiprone, a number of options are available. The dose of deferiprone may be increased from the standard 75 mg/kg/d to 100 mg/kg/d. Alternatively, deferiprone given daily, may be combined with DFO 5 days per week. Such measures resulted in a decrease in serum ferritin in all patients previously failing to respond to standard deferiprone treatment /55/. The effect of combined DFO and deferiprone on urinary iron excretion appeared to be additive, and no toxic side-effects have been observed. Improved results were also reported following alternate use of deferiprone and DFO /56/

*Deferiprone hepatotoxicity*:

Most studies of deferiprone have found fluctuations in ALT levels, particularly in the first months of treatment. The International Cooperative Group /39/ found at least one serum ALT level greater than twice the upper limit of normal in 50 of 84 patients during the first 6 months of therapy. The changes in liver enzymes were mild and transient among the 38 hepatitis C negative patients and persistent elevation occurred in only one patient, returning to pre-treatment levels when deferiprone was discontinued /39/. In the first year of the large prospective multi-center trial, mean ALT levels rose significantly at 3 and 6 months and in the intention to treat analysis, at nine months. At 12 months, however, the ALT levels did not differ significantly from baseline values /50/. Two patients discontinued therapy in the first year because of increased ALT levels. Trend analysis of ALT levels after 4 years showed no change during therapy with deferiprone, irrespective of hepatitis C status. In the study of Ceci *et al.* /57/, ALT levels did not increase among the 151 patients who completed 3 years of treatment although in those with an initial ferritin over 4000 μg/l, mean ALT levels showed a trend for decrease over time. Olivieri *et al.* reported reduction in ALT levels in most patients receiving deferiprone /43/. There are no published reports of liver failure during therapy with deferiprone.

The issue of deferiprone-associated liver injury has been particularly contentious since the report of accelerated liver fibrosis in patients receiving deferiprone in one study /46/. Of 19 thalassemic patients on long-term deferiprone treatment, 14 could be evaluated for progressive fibrosis on serial liver biopsies. Five patients receiving deferiprone were considered to have progression of fibrosis as compared to none in the retrospectively chosen group of 12 patients treated with deferoxamine. The authors estimated that the median time to progression of fibrosis was 3.2 years and the estimated odds for progression of fibrosis were 5.8 for each additional year of deferiprone treatment. As pointed out in an accompanying editorial /58/, the study was not a comparison of patients randomly assigned to either treatment, and the mean baseline hepatic iron concentration was much higher in the deferiprone (81umol/gram wet weight) than the deferoxamine (35umol/gram wet weight) group. The median age of the deferiprone group was 18.2 years compared to 13.9 years in the deferoxamine group. Likewise, the median age of the 5 patients considered to have progression of hepatic fibrosis was 21 years compared with 16 years in patients with no progression of hepatic fibrosis. Four of the five patients believed to have progression of fibrosis had antibodies to hepatitis C compared with only 2 of the 9 without progression. Patients with hepatitis C and increased liver iron greater than 7mg/g dry weight have subsequently been shown to have progression of hepatic fibrosis after bone marrow transplantation in the absence of transfusion requirement and without any chelating therapy /59/. On the other hand, in the absence of hepatitis C, only levels greater than 15mg/g dry weight were associated with progressive fibrosis. An additional limitation of the study by Olivieri *et al.* /46/ was the inclusion of biopsy specimens with as few as two portal tracts. When the same biopsies were reviewed in a blinded manner but excluding specimens with insufficient numbers of portal tracts, no progression of liver fibrosis was found in the patients treated with deferiprone /60/.

The description of increased rates of iron accumulation and hepatotoxicity in iron-loaded gerbils following treatment with diethyl-3-hydroxypyrid-4-one (CP94) /61/, a compound closely related to deferiprone /62,63/, was identified as evidence supportive of the hepatotoxicity and fibrogenic effect of deferiprone /46,64/. However, CP94 (diethyl-3-hydroxypyrid-4-one) and deferiprone (dimethyl-3-hydroxypyrid-4-one) are quite different in their biological activity /65/. Moreover, gerbils in captivity are sensitive to a range of infectious diseases causing multifocal necrotizing hepatitis /66/, and they are prone to develop hepatic fibrosis in response to endotoxin lipopolysaccharides from gut bacteria /67/. In two recent studies in pathogen-free gerbils, the iron-loading protocol employed previously by Carthew failed to produce major hepatic fibrosis /68,69/. Moreover, no evidence of a paradoxical aggravation of iron toxicity by deferiprone could be shown by blinded evaluation of hepatic histology, or as judged by tissue iron concentrations and mitochondrial respiratory enzyme activity /68/.

In view of the question raised about the role of deferiprone in the development or progression of liver fibrosis, other workers have examined single and serial liver biopsy samples from patients receiving deferiprone. Berdoukas *et al* showed no significant difference in the risk of progression to fibrosis in patients taking deferiprone or deferoxamine (70). Others have reported on small groups of patients treated with deferiprone for less than 3 years, in whom serial liver biopsies have shown no evidence of fibrosis progression.

The most comprehensive study in this area has been performed by Wanless *et al.* in 56 patients with repeat liver biopsy after a median of 3.5 (mean 3.1) years of deferiprone therapy (71). Of 58 patients in the multicenter study of deferiprone [36] with initial (less than 6 months pre-treatment) liver biopsies available, 56 consented to repeat biopsy while still being treated with deferiprone. The reason for obtaining the initial liver biopsy was variable. At one center all patients underwent biopsy before entry. At three other centers, biopsies were obtained in patients with serum ferritin levels less than 2000ug/l to determine eligibility for inclusion in the deferiprone study or, as incidental biopsies at surgery. Because initial biopsies were obtained 2 to 3 years before the first suggestion of deferiprone hepatotoxicity, there is no reason to believe that there was bias in selection. The initial biopsy served as a control for each patient. A panel of 3 pathologists evaluated retrospectively 112 coded liver biopsies obtained before and after deferiprone therapy. Fibrosis was scored with the Laennec and Ishak systems. The mean number of portal tracts was 10.2 (range 2 to 39). Forty-five patients were seropositive and 11 seronegative for hepatitis C. After a mean interval of 3.1 years, there was no significant increase in fibrosis scores by either the Laennec or Ishak systems in the 11 patients seronegative for hepatitis C or in the 45 patients seropositive for hepatitis C. There was still no significant difference when analysis was limited to the 31 patients with 6 portal tracts or more. The fibrosis score increased by more than 1 level in one patient with hepatitis C and in no patient without hepatitis C.

This study representing the largest group of evaluable patients reported so far, shows no evidence of progression of liver fibrosis that may be attributed to deferiprone toxicity. Results of this major study confirm the conclusions of previous smaller groups on the absence of evidence implicating deferiprone as a cause of liver fibrosis. It is possible that the different conclusions by Olivieri *et al.* /16/ may be attributed to the difficulties involved in evaluating fibrosis in inadequately small biopsies and the limited number of patients.

### Pyridoxal isonicotinoyl hydrazone. (PIH).

PIH is a tridentate chelator with a molecular weight of 287 (Figure 3). It was introduced by Ponka *et al.* who recognized its ability to mobilize iron from $^{59}$Fe-labelled reticulocytes /72/. At physiologic pH, PIH is mainly in its neutral form which allows access across cell membranes and absorption from the gut /73/. At pH 7.4 and a ligand concentration of 1 mmol/L, the pM value of PIH is less than for DFO. It would be worthwhile, therefore, to examine whether PIH may , or may not be able to donate chelated iron to DFO /74/, to act as a "shuttle" when the two chelators are coadministered . The selectivity of PIH for iron is comparable with that of DFO.

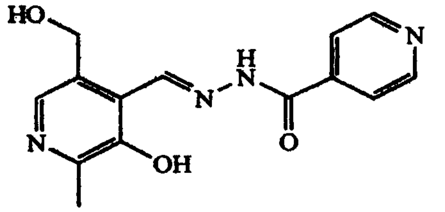

**pyridoxal isonicotinoyl hydrazone (PIH, 111)**

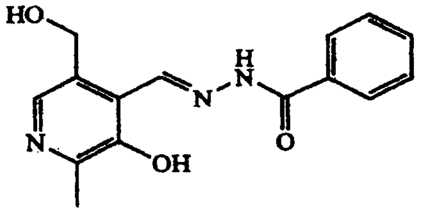

**pyridoxal benzoyl hydrazone (PBH, 101)**

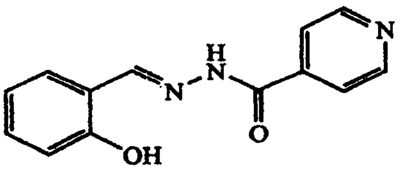

**salicylaldehyde isonicotinoyl hydrazone (SIH, 211)**

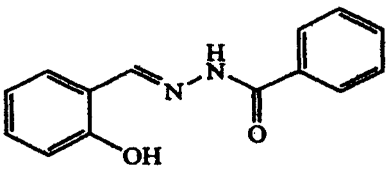

**salicylaldehyde benzoyl hydrazone (SBH, 201)**

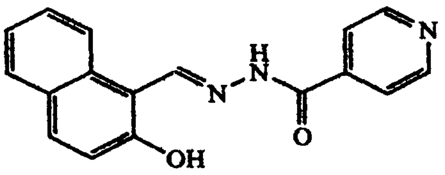

**2-hydroxy-1-naphthaldehyde isonicotinoyl hydrazone (NIH, 311)**

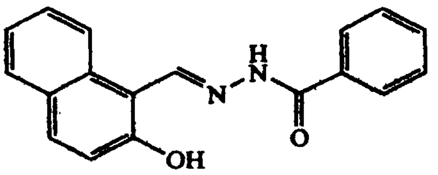

**2-hydroxy-1-naphthaldehyde benzoyl hydrazone (NBH, 301)**

**Fig. 3:** Pyridoxal isonicotinoyl hydrazone (PIH) and its analogs.

In hypertransfused rats /75/ PIH is able to remove parenchymal and RE iron with equal efficiency and practically all chelated iron is excreted through the bile. Its *in vivo* chelating efficiency is equal to, or slightly better than DFO and its oral and patenteral effectiveness is similar. No evidence of toxicity has been found at doses up to 500 mg/kg/d. However, long term (10 weeks) PIH treatment in rats failed to increase iron excretion /76/. It is possible that this failure was caused by the hydrolysis of PIH at the low pH of the stomach into pyridoxal and the corresponding acid hydrazide /77/.

Studies in patients with iron overload treated with PIH at a dose of 30 mg/kg/d have shown a modest net iron excretion of 0.12 ±0.07 mg/kg/d /78/, which is much less than the mean value of 0.5 mg/kg/d required to achieve negative iron balance in most cases. Nevertheless, it was estimated that this degree of iron excretion may be sufficient for achieving a negative iron balance in non-transfusion-dependent patients with iron loading anemias. Although the results of this pilot study in thalassemic patients were generally regarded as evidence for the limited value of PIH in the treatment of thalassemia, several arguments have been raised in favour of PIH in a recent review /79/ by Richardson and Ponka. First, the dose of 30 mg/kg used in the above study was much less than the effective doses of 125 to 500 mg/kg employed in experimental animals. Second, PIH was given to patients after calcium carbonate as a powder in gelatin capsules. This may have prevented acid hydrolysis of PIH, but also could drastically limit its absorption because of the low solubility of PIH in aqueous solution at a neutral pH /80/.

Other Schiff base compounds are readily formed with pyridoxal, and a large number of such derivatives have been studied /72,75/. One of the most effective of these, *pyridoxal- pyrimidinyl-ethoxycarbonyl methbromide* (PPH15), is able to remove 79% of hepatocellular radioiron stores in hypertransfused rats, following a single parenteral dose of 200 mg/kg, and its oral effectiveness exceeds that of parenteral DFO. Although results of preliminary toxicity studies in rats have been encouraging, subsequent studies in *Cebus* monkeys disclosed significant toxicity precluding the use of this drug for clinical purposes. A variety of PIH analogues has been prepared and evaluated by Ponka and his group /79/. One of the most promising of these is PBH (*pyridoxal-benzoyl hydrazone*). It is 280% more effective than PIH with a pK at neutral pH and 1 mmol/L concentration of 39.7 /80/. These improved features are attributed to its greater lipophilicity.

Progress in the development of PIH and its derivatives for clinical use has been painfully slow. The reasons for this have been delineated in the review of Richardson and Ponka /79/: (a) PIH is not a proprietary (patentable) agent and therefore its attractiveness for the drug industry is severely limited. (b) The interest in recent years in deferiprone has left limited space for further development of other, alternative orally efficient chelators. (c) Toxicologic and pharnacokinetic studies, although vitally important for drug development, are not sufficiently attractive to allow support by competitive governmental granting agencies. (d) On the other hand, the FDA is increasingly insistent on further toxicologic and pharmacokinetic studies as a precondition for human trials. This places the academic clinical investigator in an impossible situation. (e) To these serious limitations one may add the high likelihood that the limited apparent efficacy of PIH in the single clinical study performed so far may have discouraged further efforts in this direction. Clearly, the therapeutic potential of PIH and its derivatives still awaits extensive and careful evaluation.

### Bis-hydroxyphenyl-triazole (ICL 670A)

This compound is a member of a new class of tridentate iron-selective synthetic chelators, the bis-hydroxyphenyl-triazole produced by Novartis. ICL 670A (formerly CGP 72 670 ) *4-[3,5-bis-(hydroxyphenyl)-[1,2,4}triazol-1-yl]-benzoic acid* /81/ has been evaluated together with 44 compounds of the triazole series and about 700 other compounds of five additional chemical classes. ICL 670A has emerged from this screening process as the best compound combining oral effectiveness and low toxicity. ICL 670A has an affinity for ferric iron which is 3 log units higher than deferiprone and the ligand is stable both *in vivo* and *in vitro*.

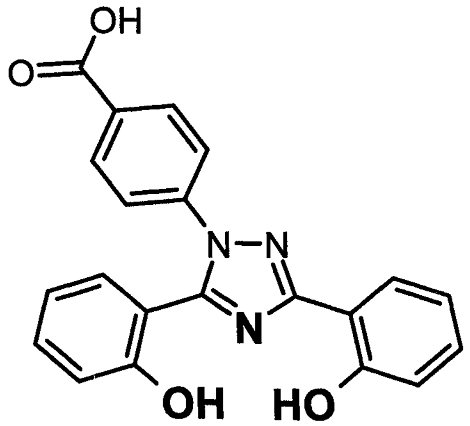

**Fig. 4:** 4-[3,5-bis-(hydroxyphenyl)-[1,2,4}triazol-1-yl]-benzoic acid - ICL670A

In a 12 week study in iron-loaded rats, ICL 670A was twice as effective orally as s.c. DFO. In the iron-loaded marmoset, iron excretion was predominantly fecal. At the higher dose range the effect of a single dose continued up to 48 hours. Iron excretion was predominantly fecal and iron-selective, without increasing copper or zinc excretion. At equimolar doses, it was 10 time more effective than deferiprone. At the effective oral dose of 22 mg/kg no adverse effects were seen after 4 weeks of treatment. At very high doses, nephrotoxicity was seen in both rats and marmosets, but this was much less in iron-loaded animals.

Phase 1 clinical trials with ICL 670A are in progress. In these studies, the safety and efficacy of. ICL 670A will be evaluated in thalassemic patients starting with a dose of 2.5 mg/kg and gradually escalating to 80 mg/kg. These studies will also include detailed pharmacokinetics including area under the curve (AUC), $C_{max}$, $t_{max}$ and $t_{1/2}$. These studies will be followed by repeated-dose safety trials and iron-balance metabolic studies /82/.

### Coadministration of chelators: the"Shuttle Effect".

Observations on the comparative ability of DFO and a hydroxypyridinone chelator (CP94) to cross cellular membranes are pertinent to the issue of probable differences in the chelatable iron pools interacting with DFO and deferiprone /83/. These studies, conducted in the rat visceral yolk sac, showed that hydroxypyridones cross the plasma and lysosomal membranes rapidly, whereas DFO is incapable of crossing the same membranes, and enters cells only by endocytosis. These *in vitro* studies lend strong support to the concept that DFO and deferiprone appear to tap different iron pools, and that their concurrent use may result in an additive, or synergistic effect on iron excretion

The combination of a weak chelator which has a better ability to penetrate cells, with a stronger chelator that penetrates cells poorly but has a more efficient urinary excretion, may result in a synergistic effect through iron shuttling between the two compounds. Such a "shuttle effect" was first proposed by Grady. Metabolic balance studies performed by Grady *et al.* in thalassemic patients have shown that, when deferiprone is given during DFO treatment (at time 0,4 and 8 of an 8-hour infusion), a synergistic effect is achieved, with total iron excretion 2.4 to 3.4 times higher than with DFO alone /84/. These data suggest an interaction between deferiprone and DFO, and may have important implications for the design of new strategies in iron chelating treatment.

A shuttle effect was directly demonstrated by following the fate of chelated plasma iron in thalassemic patients receiving combined DFO and deferiprone therapy /85/. Deferiprone treatment resulted in the temporary accumulation of chelated iron in the plasma peaking at 2 hours. The addition of DFO to deferiprone treatment resulted in the transfer of chelated iron from deferiprone to DFO and an increase in total chelated iron in the serum. This chain of events indicates improved *in vivo* chelating efficiency utilizing chelatable iron first mobilized by deferiprone, and transferred subsequently to DFO /86/.

Improved chelating efficiency and improved compliance with combined deferiprone and DFO regimen has been reported by several groups. Wonke *et al.* were the first to describe the effect of combining DFO with deferiprone treatment /87/ in thalassemic patients. Deferiprone was given daily, and DFO 5 days per week. This has resulted in a decrease in serum ferritin in all 13 patients previously failing to respond to standard deferiprone treatment. The effect of combined DFO and deferiprone on urinary iron excretion appeared to be additive, and no toxic side-effects have been observed. Combined therapy reduces serum ferritin in patients who had previously failed to achieve a satisfactory response to deferiprone alone. This approach to chelation therapy may be an attractive option for those patients who are unable to comply with deferoxamine infusions on more than a few days a week and who have an inadequate reduction of iron stores with deferiprone alone. To date, this combination therapy has shown no unanticipated side effects when given for periods of a year or more /88-91/.

It is possible that coadministration of deferiprone and DFO will achieve nothing more than the combination of their separate effects. However, several possible additional benefits are worth exploring: (a) Since the toxic side-effects of the two compounds are different and some of them are dose-dependent, cotreatment may allow dose reduction and diminishing drug toxicity. (b) Decreasing the chelatable iron pool by DFO may shift the proportion of chelatable iron to deferiprone closer to the optimal 1:3 molar ratio, allowing optimal conditions for deferiprone effect. (c) DFO has a limited access to RE iron stores but may be able to interact indirectly with RE iron by the shuttling effect of deferiprone. Such an interaction may prevent the production of the toxic NTBI originating from RBC hemoglobin breakdown in the RE system. Finally: (d) if the shuttling concept is validated, it may be utilized for the coadministration of other drug combinations. For example, Grady *et al.* have already shown that the coadministration of deferiprone and HBED greatly enhances the oral effectiveness of HBED /92/. (e) Likewise, it would be worthwhile to re-examine the clinical effectiveness of PIH (see above), or dihydroxybenzoic acid (DHB) which are weak oral chelators unable by themselves to induce a negative iron balance, in the context of combined chelating treatment with DFO. (f) *In vitro* observations on the antimalarial effect of iron chelators suggest that coadministration of lipohilic chelators with an easy access to the intracellular parasite, with extracellular DFO, may result in a synergistic parasiticidal effect /93/. This approach could be utilized in clinical studies of iron chelators in malaria.

### CURRENT ISSUES IN CHELATOR DEVELOPMENT

Undoubtedly, DFO remains the drug of choice for the management of transfusional siderosis in thalassemic patients. However, its high cost and the inconvenience of its parenteral administration by portable pumps are major limitations underlying the need for developing alternative orally effective new iron chelating drugs. In the search for new and improved chelators, it is useful to remember some of the basic principles determining the safety and efficacy of iron chelators as defined in an important review by Hider *et al.* /93/:

(a) *Stability of the chelator-iron complex*: This is determined not only by the affinity of the drug to iron(III), but also by the nature of their interaction. Hexadentate chelators bind iron at a 1:1 ratio forming a neutral, stable complex which prevents iron from participation in harmful reactions producing hydroxyl radicals. By contrast, bidentate chelators such as L1 require 3 molecules to one ferric iron to form a stable, neutral complex /94/. Hence, at suboptimal concentrations of L1, when either tissue iron concentrations are very high or drug concentrations too low, incomplete 1:1 or 2:1 complexes may be formed which may result in enhanced mobilization of potentially toxic iron. Thus, hexadentate chelators would, on theoretical grounds, be generally preferable to bidentate or tridantate chelators.

(b) *Partition coefficient*, i.e. the relative solubility in water and lipids, determines the ability to cross lipid membranes. Chelators with partition coefficients of 0.05 to 1.0 will have efficient gastrointestinal absorption. Increasing lipophilicity will result in efficient drug clearance from the portal circulation by the liver. Unfortunately, increasing lipohilicity also improves the penetration of the blood-brain and placental barriers and increases drug toxicity /95/.

(c) *Molecular weight* is a critical feature in designing new chelators with improved oral efficacy. New chelators, with excellent *in vitro* interaction with chelatable iron in cell cultures or following their parenteral administration, may be ineffective if their size interferes with their intestinal absorption. To achieve greater than 70% absorption, the molecular weight should be less than 300. Thus, by virtue of their lower molecular weights, bidentate and tridentate ligands are predicted to have higher absorption efficiency than hexadentates.

(d) *Prodrugs* are an excellent solution for a number of requirements in the development of oral chelators. Lipophilic esters represent an attractive model of non-toxic orally effective iron chelators. Such non-polar prodrugs would be efficiently absorbed and subsequently cleared from the portal system by the liver. Following conversion into the active hydrophilic metabolite, they may chelate liver iron *in situ* to be excreted in the bile or, following release into the systemic circulation, would interact with non-transferrin plasma iron and excreted in the urine. Because of their gradual absorption, iron excretion may be improved several-fold. Protection against the harmful effects of circulating NTBI is optimal when the chelator is permanently present in the plasma. This effect, similar to continuous *s.c.* or *i.v.* drug infusion, may be achieved conveniently by employing orally effective prodrugs in slow-release preparations.

(e) *Coadministration* or alternating use of DFO and a suitable oral chelator may allow a decrease in dosage of both drugs and improve compliance by decreasing the demand on tedious parenteral drug administration. Combined use of DFO and L1 has already been shown to result in successful depletion of iron stores in patients previously failing to respond to single drug therapy, and to lead to improved compliance with treatment. It may also result in a "shuttle effect" between weak intracellular chelators and powerful extracellular chelators or exploit the entero-hepatic cycle to promote fecal iron excretion. All of these innovative ways of chelator usage are now awaiting evaluation in experimental models and in the clinical setting. The introduction of the promising new chelators and the evolution of improved strategies of iron chelating therapy require better understanding of the pathophysiology of iron toxicity and the mechanism of action of iron chelating drugs.

### REFERENCES

1. Hershko C, Rachmilewitz EA. *Brit J Haemat* **42,** 125 (1979)
2. Hershko C, Graham G, Bates GW Rachmilewitz EA. *Brit J Haemat* **40,** 255 (1978)
3. Gutteridge JMC, Rowley DA, Griffiths E, Halliwell B. *Clin Sci* **68**, 463 (1985)
4. Al-Refaie FN, Wickens DG, Wonke B, Kontoghiorghes GJ, Hoffbrand AV. *Brit J Haemat* **82**, 431 (1992)
5. Porter JB, Abeysinghe RD, Marshall L, Hider RC, Singh S. Blood **88**, 705 (1996)
6. Link G, Pinson A, Hershko C. *J Lab Clin Med* **106**, 147 (1985)
7. Randell EW, Parkes JG, Olivieri NF, Templeton DM. *J Biol Chem* **269**, 16046 (1994)
8. Link G, Athias P, Grynberg A, Pinson A, Hershko C. *J Lab Clin Med* **113**, 103 (1989)
9. Gabutti V, Borgna-Pignatti C. *Clin Haemat* **7**, 919 (1994)
10. Link G, Pinson A, Hershko C. Blood **83,** 2692 (1994)
11. Link G, Pinson A, Hershko C. *J Lab Clin Med* **121**, 127 (1993)
12. Link G, Tirosh R, Pinson A, Hershko C. *J Lab Clin Med* **127**, 272 (1996)
13. Aldouri MA, Wonke B, Hoffbrand AV, Flynn DM, Ward SE, Agnew JE, Hilson AJ. *Acta Haematol* **83**, 113 (1990)
14. Mariotti E, Agostini A, Angelucci E. et al. *Bone Marrow Transpl* **12** (suppl 1), 14 (1993)
15. Gabutti V, Piga A. *Acta Haemat*, **95**, 26 (1996)
16. Olivieri NF, Brittenham GM. *Blood* **89**, 739-761 (1997)
17. Porter JB, Faherty A, Stallibrass L, Brookman L, Hassan L, Howes C. *Ann N Y Acad Sci* **850**, 485 (1998)
18. Borgna-Pignatti C, Cohen A. J Pediat **130**, 86 (1997)
19. Araujo A, Kosaryan M, MacDowell A, Wickens D, Puri S, Wonke B, Hoffbrand AV. *Brit J Haemat*ol **93**, 835 (1996)

20. Canatan D, Temimhan N, Dincer N, Ozsancak A, Oguz N, Temimhan M. Acta Paediatr **88**, 550 (1999)
21. Borgna-Pignatti C, Rugolotto S, De Stefano P, Piga A, Di Gregorio F, Gamberini MR, Sabato V, Melevendi C, Cappellini MD, Verlato G. *Ann N Y Acad Sci* **850**, 227 (1998)
22. Brittenham GM, Griffith PM, Nienhuis AW. et al. *New Engl J Med* **331**, 567 (1994)
23. Olivieri NF, Nathan DG, MacMillan JH, Wayne AS, Liu PP, McGee A, Martin M, Koren G, Cohen AR. *N Engl J Med* **331**, 574 (1994)
24. Giardina PJ, Grady RW, Ehlers KH, Burstein S, Graziano JH, Markenson AL, Hilgartner MW. In: A. Bank, Ed. *Sixth Cooley's Anemia Symposium, Ann N Y Acad Sci* **612**, 275 (1990)
25. Hoffbrand AV, Wonke B. *Bailliere's Clin Haemat* **2**, 345 (1989)
26. Calleja EM, Shen JY, Lesser M, Grady RW, New MI, Giardina PJ. *Ann N Y Acad Sci* **850**, 469 (1998)
27. Marcus RE, Davies SC, Bantock HM, Underwood SR, Walton S, Huehns ER.. *Lancet* **1**, 392 (1984)
28. Hyman CB, Agness CL, Rodriguez-Funes R,. Zednikova M. *Ann N Y Acad Sci* **445**, 293 (1985)
29. Cohen AR, Martin M, Schwartz E. In: A. Bank, Ed. Sixth Cooley's Anemia Symposium, *Ann N Y Acad Sci* **612**, 286 (1990)
30. Davis BA, Porter JB. *Blood.* **95**, 1229 (2000)
31. Hider RC, Singh S, Porter JB, Huehns ER. In: A. Bank, Ed. *Sixth Cooley's Anemia Symposium, Ann N Y Acad Sci* **612**, 327 (1990)
32. Kontoghiorghes GJ. In: A. Bank, Ed. *Sixth Cooley's Anemia Symposium, Ann N Y Acad Sci* **612**, 339 (1990)
33. Diav-Citrin O, Koren G. *Pediat Clin North Amer* **44**, 235 (1997)
34. Barman Balfour JA, Foster RH. *Deferiprone. Drugs.* **58**, 553 (1999)
35. Olivieri NF. *Acta Haematol* **95**, 37 (1996)
36. Al-Refaie FN, Hoffbrand AV. *Clin Haemato*l **7**, 941 (1994)
37. Grady RW, Srinivasan R, Dunn JB, Coursey MP, Lemert RF, Calvano SE, Hilgartner MW. *Drugs Today*, Suppl. A. **28**, 73 (1992)
38. Ziel R, Junker Walker U, Pfaar U, Vogel B, Bentley P, Schnebli HP. *5th International Conference on Thalassaemias and the Haemoglobinopathies.* Nicosia, Cyprus. : Abstract #191 (1993)
39. Al-Refaie FN, Hershko C, Hoffbrand AV, Kosaryan M, Olivieri NF, Tondury P, Wonke B. *Brit J Haemat* **91**, 224 (1995)
40. Vallee BL, Falchuk KH. *Physiol Rev* **73**, 79 (1993)
41. Berdoukas V, Bentley P, Frost H, Schnebli HP. *Lancet* **341**, 1088 (1993)
42. Hershko C. *Lancet* **341**, 1088 (1993)
43. Olivieri NF, Brittenham JM, Matsui D, Berkovitch M, Blendis LM, Cameron RG, McLelland RA, Liu PP, Templeton DM, Koren G. *N Engl J Med* **332**, 918 (1995)
44. Tondury P, Kontoghiorghes GJ, Ridolfi-Luthy A, Hirt A, Hoffbrand AV, Lottenbach AM, Sonderegger T, Wagner HP. *Brit J Haemat* **76**, 550 (1990)
45. Agarwal MB, Gupte SS, Viswanathan C, Ramanathan J, Desai N, Puniyani RR, Chhablani AT. *Brit J Haemat* **82**, 460 (1992)
46. Olivieri NF, Brittenham GM, McLaren CE, Templeton DM, Cameron RG, McClelland RA, Burt AD, Fleming KA. *New Engl J Med* **339**, 417 (1998)

47. .47. Olivieri NF, Brittenham GM. *Blood* Suppl. 1. **90**, Abstract # 1161 (1997)
48. Hoffbrand AV, Al-Refaie F, Davis B, Siritanakathul N, Jackson BFA, Cochrane J et al. *Blood* **91**, 295 (1998)
49. Tondury P, Zimmermann A, Nielsen P, Hirt A.. *Brit J Haematol*. **101**, 413 (1998)
50. Cohen AR, Galanella R, Piga A, DiPalma A, Vullo C, Tricta F. *Br J Haematol.* **108**, 305 (2000)
51. Castriota-Scanderberg A, Sacco M. *Brit J Haematol*. **96**, 254 (1997)
52. Addis A, Loebstein R, Koren G, Einarson TR. *Eur J Clin Pharmacol* **55**, 1 (1999)
53. Tahr A, Chamoun FM, Koussa S,aad MA, Khoriaty AI, Neeman R, Mourad FH. *Acta Haematol* **101**, 173 (1999)
54. Grady RW, Hilgartner MW, Giardina PJ. *Blood* Suppl. 1. **88**, Abstract # 1230 (1996)
55. Wonke B, Wright C, Hoffbrand AV. *Brit J Haematol* **103**, 361 (1998)
56. Aydinok Y, Nisli G, Kavakli K. *Brit J Haematol* **106**, 252 (1999)
57. Ceci A, Baiardi P, Felisi M et al. *Brit J Haematol* 2002; **118**, 330 (2002)
58. Kowdley KV, Kaplan MH. *N Engl J Med.* **339**, 468 (1998)
59. Angelucci E, Muretto P, Nicolucci A et al. *Blood.* **100**, 17 (2002)
60. Callea F. *N Engl J Med.* **339**, 1709 (letter) (1998)
61. Carthew P, Smith AG, Hider RC et al. *Biometals.* **7**, 267 (1994)
62. Hider RC, Singh S, Porter JB, Huehns ER. In: Bank A, ed *Sixth Cooley's Anemia Symposium*, *Ann N Y Acad Sci.* **95**, 6 (1990)
63. Hider RC, Choudhury R, Rai BL et al. *Acta Haematol.* **95**, 6 (1996)
64. Olivieri NF. Hematology 2001. *Am Soc Hematol Educ Prog Book.* 521 (2001)
65. Porter JB, Abeusinghe RD, Hoyes KP et al. *Brit J Haemat.* **85**, 159 (1993)
66. Schoeb TR. Disease of Gerbils. PAT 707, Pathology of Laboratory Animals II. Winter Quarter 1989-1990. Taxonomy. Dept. Comparative Med U of Alabama, Birmingham. hhtp://netvet.wustl.edu/species/gerbils (1989-1990)
67. Carthew P, Edwards RE, Dorman BM. *J Comp Pathol.* **104**, 303 (1991)
68. Hershko C, Link G, Konijn AM et al. *J Lab Clin Med.* **139**, 50 (2002)
69. Ramm GA, Britton RS, Brunt EM et al. (abstract) *Bioiron 99, World Congress, Excess Cardiac on Iron Metabolism*, Sorrento, Naples, Italy. 327a. (1999)
70. Berdoukas V, Bohane T, Eagle C et al. *Transf Sci.* **23**, 239 (2000)
71. Wanless A, Sweeney G. Dhillon AP et al. *Blood.* **100**, 1566 (2002)
72. Ponka P, Borova J, Neuwirth J, Fuchs O, Necas E. *Biochim Biophys Acta* **586**, 278 (1979)
73. Richardson DR, Baker E. *Biochim Biophys Acta* **1091**, 294 (1991)
74. Ponka P, Schulman HM, Wilczynska A. *Biochim Biophys Acta* **718**, 151 (1982)
75. Hershko C, Avramovici-Grisaru S, Link G, Gelfand L, Sarel, S. *J Lab Clin Med* **98**, 99 (1981)
76. Williams A, Hoy T, Pugh A, Jacobs A. *J Pharm Pharmacol* **34**, 730 (1982)
77. Richardson DR, Wis Vitolo L, Baker E, Webb J.**2**, 69 (1989)
78. Brittenham GM. *Sein Hematol* **27**, 112 (1990)
79. Richardson DR, Ponka P. *J Lab Clin Med* **131**, 306 (1998)
80. Vitolo LMW, Hefter GT, Clare BW, Webb J. *Inorg Chim Acta* **170**, 171 (1990)

81. Acklin P, Lattmann R, Buhlmayer P, Crowe A, Faller B, Jin Y, Nick HP, Sergejew T, Spanka C, Wong A, Zbinden P, Schnebli HP. *Chimia* **52**, 446 (1998)
82. Galanello R, Loehrer F, Alberti D, Piga A. *The 7th International Conference on Thalassaemia and the Haemoglobinopathies*. Bangkok, Thailand, Abstract S1704, p189 (1999)
83. Cable, H, Lloyd JB. *J Pharm Pharmacol.* **51**, 131 (1999)
84. Grady RW, Berdoukas VA, Rachmilewitz EA, Giardina PJ. *The 7th International Conference on Thalassaemia and the Haemoglobinopathies*. Bangkok, Thailand, Abstract 0018 (1999)
85. Breuer W, Empers MJJ, Pootrakul P, Abramov A, Hershko C, Cabantchik I. *Blood.* **97**, 792(2001)
86. Link G, Konijn AM, Breuer W, Cabantchik I, Hershko C. *J Lab Clin Med.* **138**, 130 (2001)
87. Wonke B, Wright C, Hoffbrand AV. *Br J Haematol.* **103**, 361 (1998)
88. Balveer K, Pryor K, Wonke B. *Med J Malaysia.* **55**, 493 (2001)
89. Taher A, Mourad FH, Shackh-Taha M et al. *11th International Conference on Oral Chelation in the Treatment of Thalassemia and Other Diseases*. Catania. 144 (2001)
90. Farmaki K, Anagnostopoulos G, Platis O, Gotsis E, Toulas P. *The Hematology Journal* **3**, Suppl. 1;79a (2002)
91. Galanello R, Doneddu I, Dessi E et al. *11th International Conference on Oral Chelation in the Treatment of Thalassemia and Other Diseases*. Catania 73a (2001)
92. Grady RW, Giardina PJ. *Blood* **94**, Suppl. 1 abstract 3293 (1999)
93. Cabantchik ZI, Glickstein H, Golenser J, Loyevsky M, Tsafack A. *Acta Haemat* **9**, 70 (1996)
94. Hider RC, Choudhuri R, Rai BL, Dehkordi LS, Singh S. *Acta Haematol* **9**, 6 (1996)
95. Porter JB, Morgan J, Hoyes KP, Burke LC, Huehns ER, Hider RC. *Blood* 7, 2389 (1990)

